# Copolymers enhance selective bacterial community colonization for potential root zone applications

**DOI:** 10.1038/s41598-017-16253-0

**Published:** 2017-11-21

**Authors:** Vy T. H. Pham, Pandiyan Murugaraj, Falko Mathes, Boon K. Tan, Vi Khanh Truong, Daniel V. Murphy, David E. Mainwaring

**Affiliations:** 1 School of Science, Faculty of Science, Engineering and Technology, Swinburne University of Technology, Hawthorn, VIC 3122 Australia; 2 SoilsWest, UWA School of Agriculture and Environment, Faculty of Science, The University of Western Australia, Crawley, WA6009 Australia

## Abstract

Managing the impact of anthropogenic and climate induced stress on plant growth remains a challenge. Here we show that polymeric hydrogels, which maintain their hydrous state, can be designed to exploit functional interactions with soil microorganisms. This microbial enhancement may mitigate biotic and abiotic stresses limiting productivity. The presence of mannan chains within synthetic polyacrylic acid (PAA) enhanced the dynamics and selectivity of bacterial ingress in model microbial systems and soil microcosms. *Pseudomonas fluorescens* exhibiting high mannan binding adhesins showed higher ingress and localised microcolonies throughout the polymeric network. In contrast, ingress of *Bacillus subtilis*, lacking adhesins, was unaltered by mannan showing motility comparable to bulk liquids. Incubation within microcosms of an agricultural soil yielded hydrogel populations significantly increased from the corresponding soil. Bacterial diversity was markedly higher in mannan containing hydrogels compared to both control polymer and soil, indicating enhanced selectivity towards microbial families that contain plant beneficial species. Here we propose functional polymers applied to the potential root zone which can positively influence rhizobacteria colonization and potentially plant growth as a new approach to stress tolerance.

## Introduction

Microbial interactions with polymeric hydrogels play a pivotal role in biomaterials, cell encapsulates and inoculants, while the interactions with biological hydrogels also represent a key component of bacterial pathogenicity^1–4^. It is widely recognised that material properties such as swelling and network rigidity influence both bacterial motility and viability^5,6^. Bacterial population and survival within the plant rhizosphere represents an area of critical importance during the increasing periods of environmental (climatic) stress^7,8^. A wide range of chemical functionalities show an ability to influence bacterial adhesion and cell signalling^9,10^, while surface stiffness, as a material property, significantly influences bacterial attachment and growth^11,12^ which may be extended to stress tolerance during colonization.

The hydrodynamics of microorganism swimming in both the liquid state and across hydrated surfaces has gained research prominence^6,13^, particularly motility at the microscale where only viscous drag is available for propulsion^14^. Motility in polymeric media imposes non-Newtonian rheology on propulsion and bacterial behaviour such as self-organization. Here, polymers deform and relax under shear stress, which at the microscale of motility provides imbalanced forces compared to aqueous Newtonian fluids^14,15^, which produce microbial swimming trajectories unlike the bulk or adjacent rigid surfaces^16^. Notably, copolymers and inter-penetrating polymer networks provide a confining environment on the scale of these hydrodynamic interactions, as well as a spatial isolation shown to influence microbial diversity within aqueous pockets of unsaturated soil through nutrient diffusion limitations^17–19^. Thus addition of functionalized polymer hydrogels analogous to inoculants, represent an area with potential to alleviate both biotic and abiotic stresses and thereby increase crop production.

Protein-carbohydrate interactions play a significant role in bacterial adhesion and infection involving both animal and plant cells^20–22^. Free multivalent carbohydrates mediate bacterial adhesion by preferentially binding to bacterial adhesin proteins (*e.g*. oligomannoses and lectin) associated with pili or fimbriae on Gram-negative cell surfaces^20,22,23^. This was observed within the rhizosphere where recognition establishes close contact with root cells enhancing interactions and colonization of rhizobial bacteria such as the Proteobacteria family^24,25^.

Previously, we detailed microbial ingress into the confined volume of synthetic polyacrylic acid (PAA) hydrogel networks in terms of the dynamic advancing population front and self-organization into well defined clusters and micro-colonies^26^. Here, we investigate how the presence of mannan biopolymer chains, capable of specific interactions with bacterial adhesin proteins, influences the microbial population when they form crosslinked copolymers or free interpenetrating networks (IPNs). Limiting imaging to low bacterial densities and short incubation times, allowed identification of bacterial motility and self-organization within the confined fluid phase of the hydrogels. The influence of mannan chains now clearly demontrates microbial selectivity in bacterial communities when in contact with a soil. Small quantities of copolymers, in close proximity to developing root systems, suggest new pathways for the native biota to provide enhanced tolerance during increasing water stress, which is further enhanced by their ability to maintain a hydrous state during soil drying periods.

## Results and Discussion

### Production and characterization of hydrogel systems

**Figure 1:**
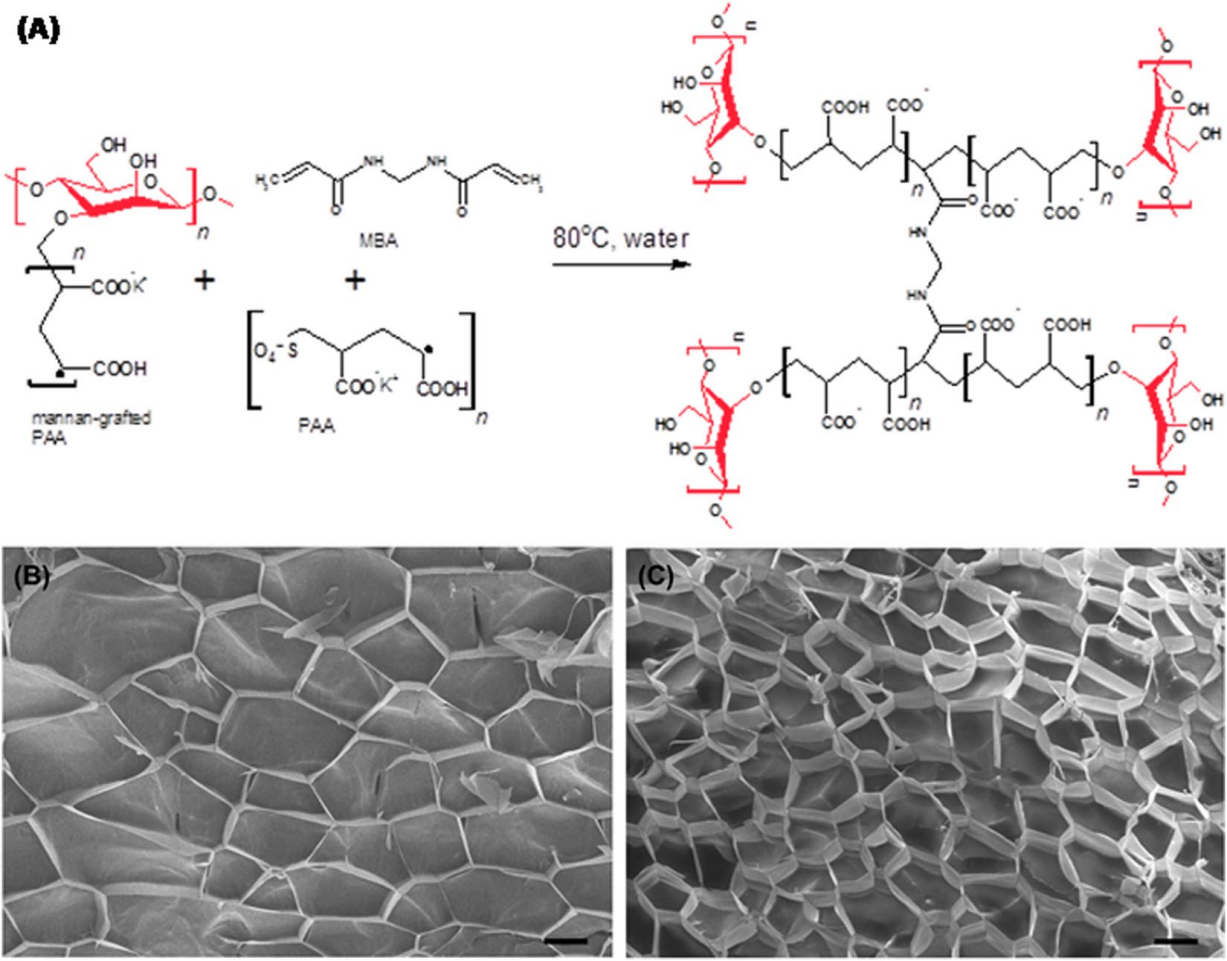
Mesoporous structure of swollen hydrogels. (**A**) Proposed free radical polymerization mechanism showing mannan attached to polyacrylic acid or K-polyacrylate (PAA) chains and further PAA-mannan grafting to form the cross-linked hydrogel matrix; (**B**) The mesoporous network of PAA and (**C**) PAA-mannan imaged by cryo-SEM (scale bar 20 µm).

Polyacrylic acid (PAA) was prepared by free radical crosslinking^27^ (Supplementrary Information S1), while the reaction scheme was further extended to yield the grafted PAA-mannan (PAA-mann_graft_) copolymer through crosslinking between free radicals generated on both the acrylate π bond and mannan hydroxyl groups (Fig. 1A and Supplementary Fig. 1). The corresponding mannan inter-penetrating polymer hydrogel (PAA-mann_free_) was prepared from PAA by physical absorption from mannan solution. Incorporation of the mannan chains as a grafted copolymer yields a hydrogel suggesting a similar mesoporous microstructure to synthetic PAA (approximately 50 µm in diameter)^26,28^, although the cell size of PAA-mann_graft_ was on average ~50% smaller (Fig. 1B,C). Estimation of the cell wall thicknesses of both PAA and its copolymer were similar (~1 µm), indicating the similar extent of confined free liquid within the system potentially available for microbial transport. Mannan grafting significantly decreases the rigidity of the gel network as seen by the yield stress at comparable degrees of swelling (Q) as well as reducing the corresponding storage (G’) and viscous (G”) moduli, reflecting the reduced crosslink density between PAA chains, both of which have implications for overall bacterial population dynamics^16^ (Supplementary Fig. 2).

### Dynamics of bacteria ingress within hydrogels

**Figure 2:**
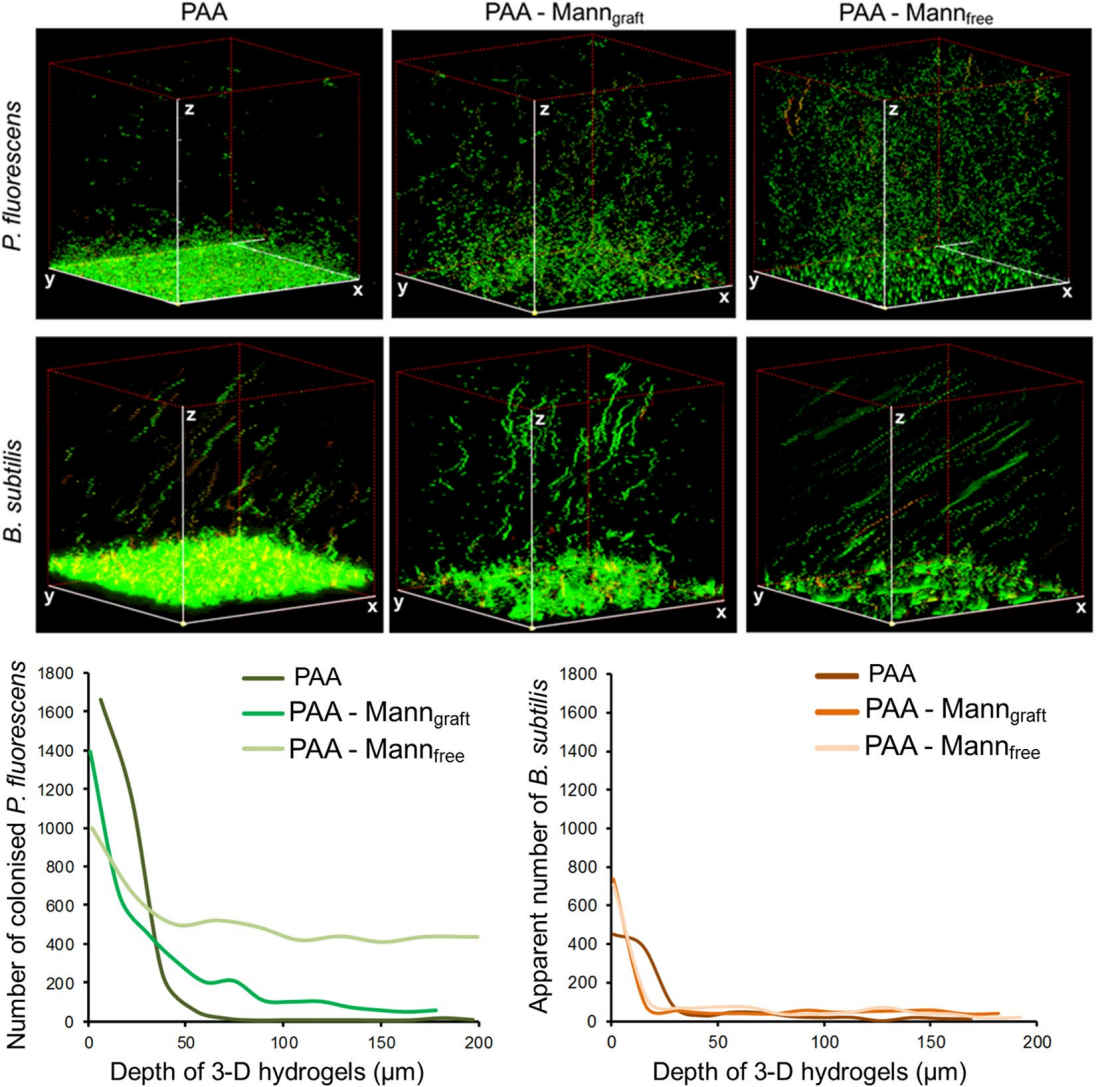
Bacterial ingress in hydrogels over 18 hour. Confocal images and population profiles showing the ingress of *Pseudomonas fluorescens* (adhesin present) and *Bacillus subtilis* (without adhesin) into the PAA polymer, grafted PAA-mannan and freely absorbed PAA-mannan after 18 hours of incubation (all hydrogels were pre-swollen in nutrient solution). Here distinct differences between the presence or absence of mannan chains in the hydrogels and adhesin binding proteins on the cells are clearly seen. The xyz imaging dimensions were 211 µm × 211 µm × 200 µm respectively. Images represent the cross-section of CLSM scans, showing the biointerface (cells enter the 3D hydrogel from xy plane) and the inner bulk regions of bacteria-colonized polymer. Green indicates viable cells (SYTO 9 stained), while red indicates damaged cells (propidium iodide stained). The number of colonized *P. fluorescens* and the apparent movement of *B. subtilis* in the 3-D structure of hydrogels were quantified using ImageJ. Differences in microbial motility and colonization are shown in Suppl. Figure 3.

Two model bacteria, with and without mannan adhesins, were selected initially to explore the influence of copolymerization with mannan on bacterial population and viability, when pre-swollen with either water or nutient broth. The PAA hydrogel as well as their mannan containing counterparts, when pre-swollen with nutient broth, show a significant difference in bacterial translocation *i.e*. the advancing population profile between the motile monotrichous *Pseudomonas fluorescens* having a single polar flagellum and the peritrichous *Bacillus subtilis* having about 14 flagella filaments^29,30^. Inspection of the 3-D fluorescence images of Fig. 2 indicates that *P. fluorescens* populates the inner bulk hydrogel region as dispersed microcolonies throughout the mesoporous network, whereas the superimposed z-stack images of *B. subtilis* indicate ingress with well defined linear movements during ingress (Supplementary Fig. 3). Comparison of PAA with its mannan counterparts clearly indicates that these differences in the underlying motility behaviour are species-specific with the mannans predominately increasing population densities. Supplementary Fig. 4 indicates that upon soil drying over prolonged periods, the presence of small quantities of PAA increases the overall soil moisture by about 10% which is retained as a free liquid phase environment within the swollen hydrogel at a wt. ratio water:PAA of 50:1.

**Figure 3:**
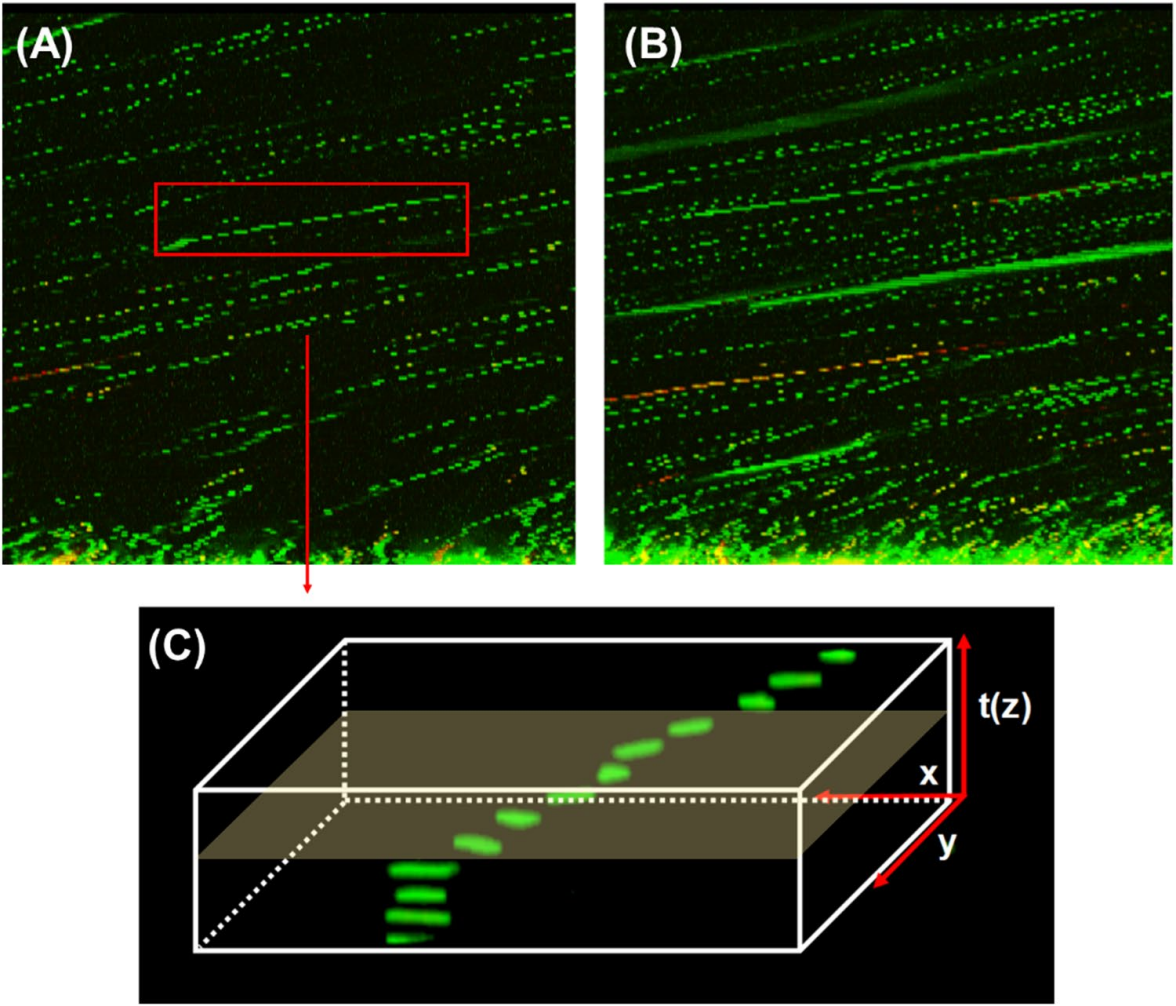
Motility of *B. subtilis* after 18 hours. (**A**) Confocal images of *Bacillus subtilis* cells ingress into PAA-mann_free_ after 18 hours, where xz dimension is 211 µm × 200 µm respectively. (**B**) Subsequent scan of same area (**A**) showing different patterns of *B. subtilis* cells, indicating that cells were motile in the bulk region of the hydrogels. (**C**) The swimming velocity of *B. subtilis* cells in the hydrogels estimated from the distance a single cell travelled and time required for capture of successive planes travelled through within the field of view. The estimated swimming velocities of *B. subtilis* in PAA and PAA-mann_free_ were 33.23 ± 4.35 µm/s and 32.44 ± 7.93 µm/s respectively.

This difference in the collective behaviour was further studied in Fig. 3 in terms of the motility of single *B. subtilis* cells. Figure 3(A,B) show two consecutive scans of different images of the same volume location demonstrating that cells were motile in the confined fluid of the polymer gel. Figure 3(C) provides the 3-D swimming trajectory in the x,y,z(time) stack. From the distance travelled by a single cell and the time required to capture each plane as it moved through the field of view, the estimated swimming velocity of *B. subtilis* was 33.2 ± 4.3 µm/s for the PAA hydrogel, which is consistent with the swimming speed of 25–30 µm/s in bulk liquids^30,31^. Cisneros *et al*. found that increasing the cell – cell proximity of *B. subtilis* increased the swimming velocity to 40 µm/s which was attributed to enhancing collective turbulance, which is not seen at the low ingress times and population densities studied here^30^. Both visual images and the quantitative analysis of total cell numbers with depth (z) (see Fig. 2) show that incorporation of the grafted and absorbed mannans reduce the *P. fluorescens* concentration in the proximity of the hydrogel surface while within the interior of the hydrogel the overall population density is significantly increased. Importantly, freely absorbed mannans increase the translocation and colonization of these bacterial species greater than grafted chains as shown by their lower surface density but higher internal population. The behaviour of *P. fluorescens* is consistent with the presence of high mannose binding adhesins which promote localization^32,33^, whereas these adhesins have been shown to be absent in the genome sequence of *B. subtilis* ^34^. The relative internal cell population in hydrogel region between 50 to 170 µm, i.e excluding the interfacial area of 0–49 µm, were 1,780, 12,385, and 65,965 cells in PAA, PAA-mann_graft_ and PAA-mann_free_ respectively. On the basis of the relative mannan contents, this represents an order of magnitude greater colonization in the presence of freely absorbed carbohydrate chains (IPN) (approximately 1:20 in average), suggesting that grafting yields less accessibile mannan chain conformations to the cell adhesins. The collective behaviour of the motile *B. subtilis* within the confined volumes of the gel was characterised by running, tumbling and cell division (Supplementary Fig. 3) consistent with the quantitative bulk motility behaviour reported by Li for this species^29^. Interestingly, the presence of free mannans absorbed within the PAA hydrogel did not influence significantly the swimming velocity of the adhesin-lacking *B. subtilis* (32.4 ± 7.9 µm/s) compared to motility in the fluid phase of the mesoporous PAA hydrogel (Fig. 3), suggesting that mannan chains are largely associated with the hydrogel polymer macropore walls (Fig. 1B). Additionally, incubation of *B. subtilis* with the hydrogels for 48 hours (Supplementary Fig. 5) indicated that the relative behaviours of both PAA and PAA-mannan were not influenced by the time duration of incubation.

**Figure 4:**
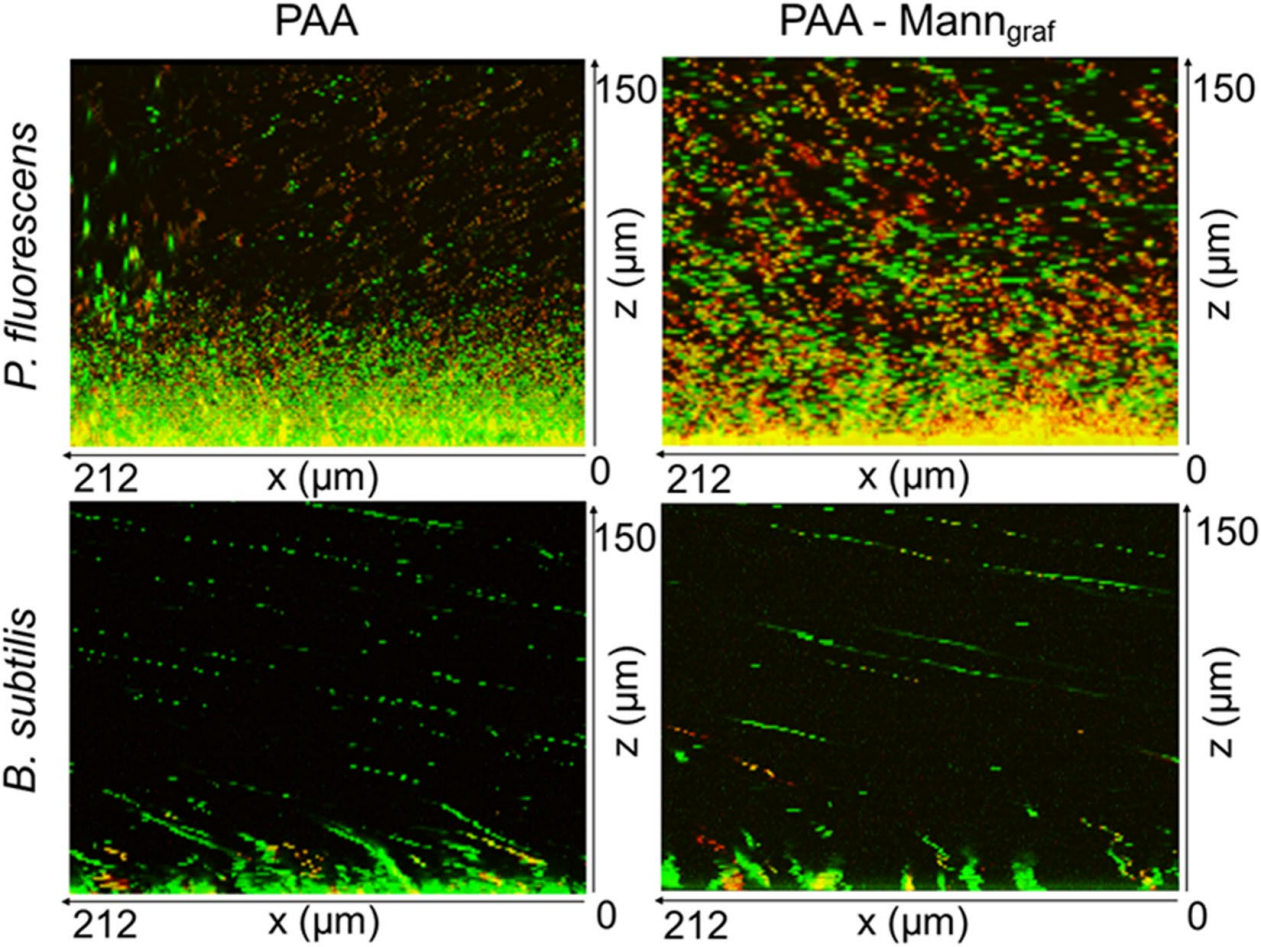
Bacterial viability in hydrogels in the absence of nutrient. Differential bacterial ingress of *Pseudomonas fluorescens* and *Bacillus subtilis* cells into PAA and grafted PAA-mannan hydrogels when swollen in water followed by the incubation with bacteria for 18 hours. When PAA-mannan was swollen in water, the bacteria exhibited low viability (yellow indicates onset of damage and red indicates substantial damage of cells), which suggests that the mannan polymer does not act as a significant nutrient source for both bacteria.

The presence of grafted mannan slightly increases the *P. fluorescens* population in the inner bulk region of the hydrogel when both were pre-swollen in water only (Fig. 4). The number of damaged and non-viable cells were also seen to increase markedly, indicating that the carbohydrate does not act as a significant nutrient source. Notably, inclusion of mannan shows little effect on the ingress of *B. subtilis* when the hydrogel was pre-swollen in water, also indicating that mannan does not act as a direct nutrient source for this species. Importantly, when all samples were swollen in nutrient broth, *P. fluorescens* still showed a significant increase in population within the PAA-mannan hydrogel, indicating the important role of mannan in enhancing the colonization of adhesin-associated *P. fluorescens*.

### Soil microbe population and taxa selectivity

**Figure 5:**
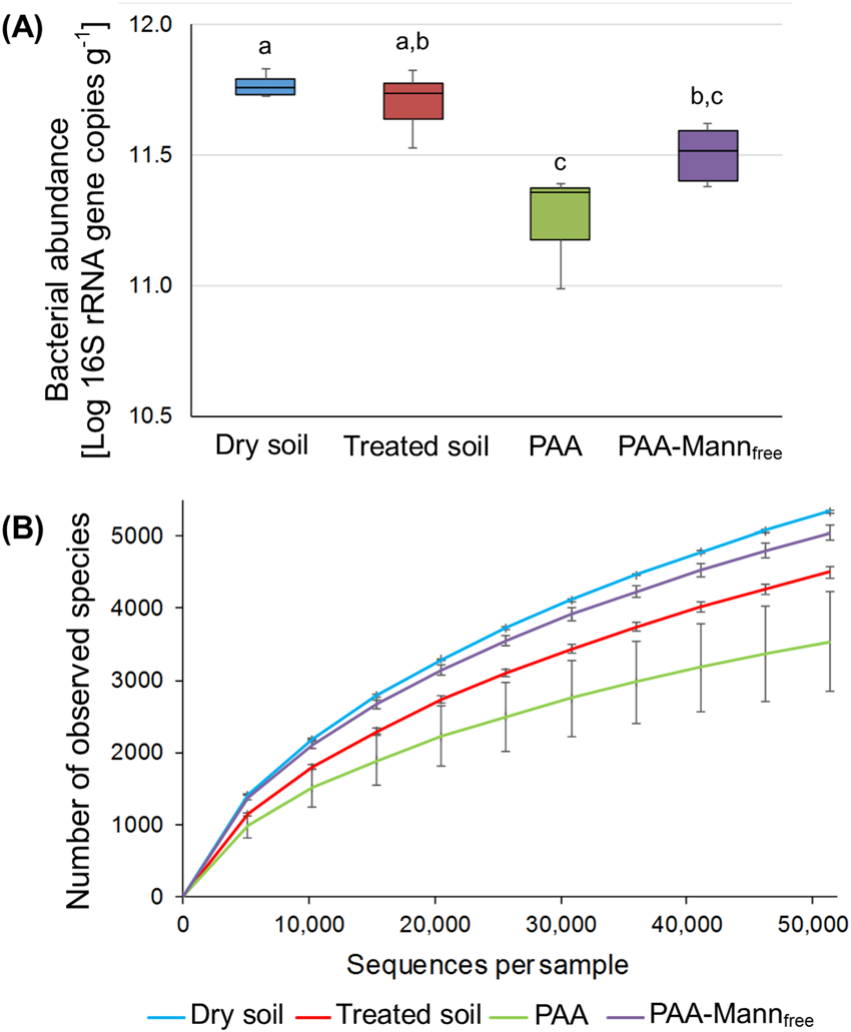
Bacterial abundance and alpha diversity in microcosms after 7 days. (**A**) Bacterial abundance within hydrogels incubated in an agricultural soil for 7 days at 25 °C compared to the original dry soil (negative control), and soil treated as above but without hydrogel (positive control), as seen by 16 S rRNA gene qPCR (n = 5, except for PAA n = 3). (**B**) The comparable number of OTUs in the respective samples (n = 5). Different letters represent statistical significant (P < 0.05) based on PRIMER-E test.

**Figure 6:**
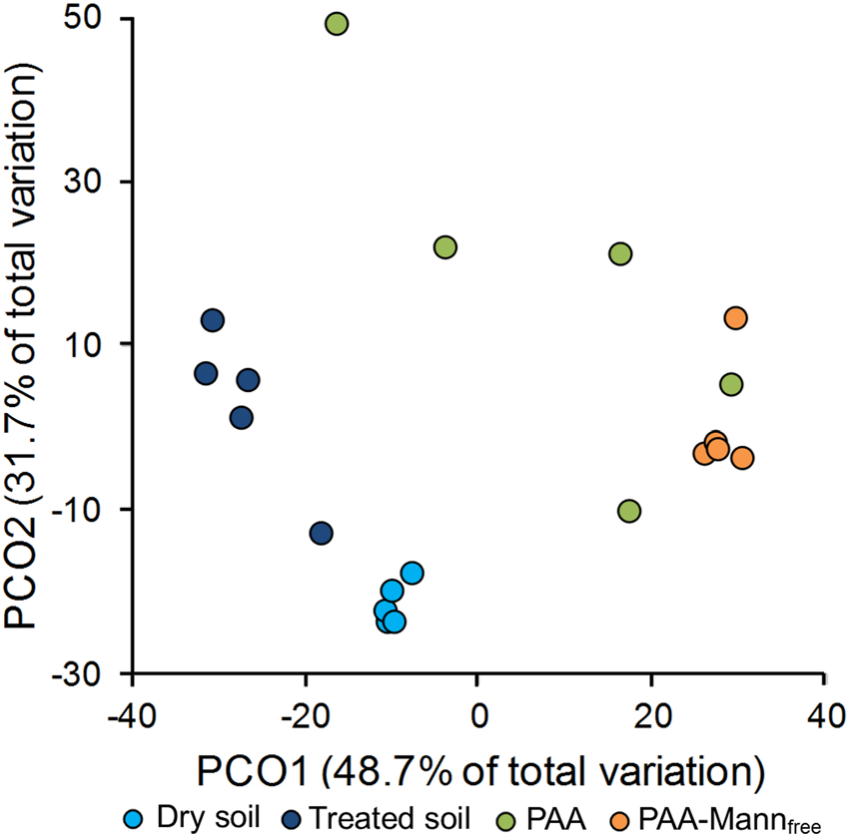
Bacterial community similarities shown by principal coordinate analysis. PCoA plot based on weighted UniFrac showing similarities in the bacterial communities between hydrogels incubated in Dandaragan soil for 7 days compared to the original dry soil (negative control) and treated soil incubated as above (positive control)^59^.

**Figure 7:**
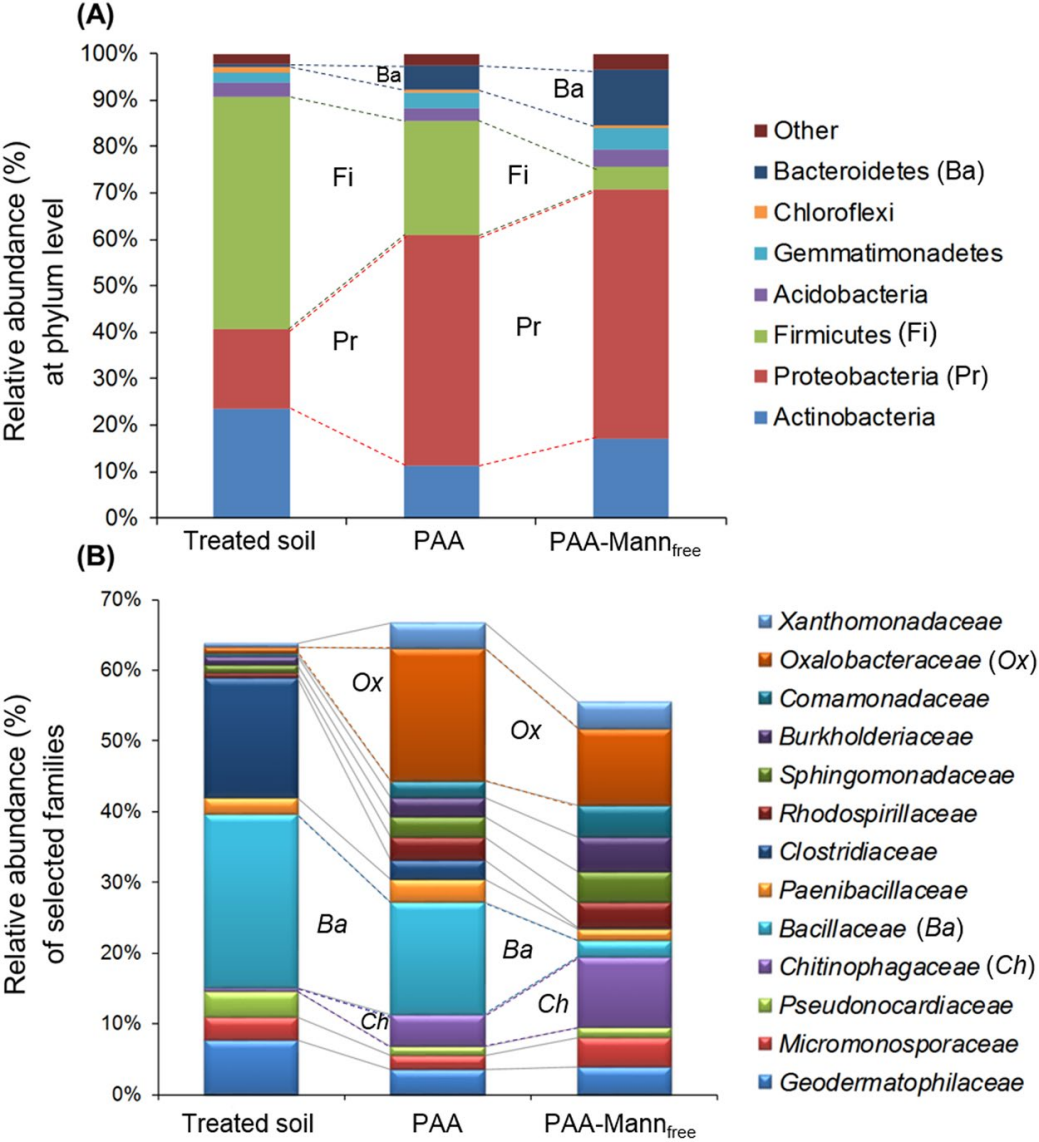
Relative compositions of bacterial community after 7 days. Relative abundance of the most representative phyla (**A**), and families identified by SIMPER analysis explaining the dissimilarities between sample types (**B**) detected in Dandaragan soil (positive control) and hydrogel microcosms shown in Fig. 5 (n = 5).

Microbial populations were investigated in soil and soil-hydrogel microcosms established from the surface horizon of a low fertility Western Australian coarse-textured agricultural soil used for wheat production. Following DNA extraction, microbial populations were identified and quantified by 16 S rRNA sequencing and qPCR respectively, where it was found that bacterial community compositions within the PAA and PAA-mann hydrogels were significantly different from the corresponding soil (positive control) after 7 days of incubation (p = 0.001; <47.8% similarity), as shown in Figs 5 and 6. Here it can be seen that the presence of mannan within the hydrogels increases the bacterial population beyond that of PAA although lower than original dry soil (negative control) and the treated soil (positive control), whereas PAA-mannan significantly increases the number of taxa present above both the treated soil and the synthetic PAA close to the original dry soil. The soil bacterial community was dominated by the phylum Firmicutes (50.1%) whereas Proteobacteria represented the most abundant group in the hydrogel polymers (49.6% in PAA and 53.7% in PAA-mann_free_ (Fig. 7A). In both hydrogel types, the family *Oxalobacteraceae* (Burkholderiales, Betaproteobacteria) constituted the most abundant family (10.9% to 18.8%, Fig. 7B) in contrast to the soil itself. Although the bacterial communities within the polymers were not significantly different from each other (p = 0.057; 60.2% similarity), the second most abundant family in PAA were the *Bacillaceae* (16.0%) which only constituted 2.3% in the PAA-mann_free_ (Fig. 7B). Whereas, the second most abundant group in the PAA-mann_free_ polymers were the *Chitinophagaceae* (Sphingobacterilaes, Bacteroidetes). Interestingly, the number of operational taxonomic units (OTUs) was significantly higher in the PAA-mann_free_ hydrogel than in the incubated soil (p = 0.006) which in turn was higher than conventional PAA (Fig. 5B). The latter may be caused by the high variance in OTU numbers in the PAA (Fig. 5B).

Due to incubation of soil close to soil water field capacity for 7 days, the community composition of the treated soil, which reflects the diversity within the local habitat e.g. soil or hydrogel, was quantitatively significantly lower than in the dry soil (p = 0.0005) which had been stored dry followed by DNA sampling before wetting (Supplementary Fig. 6A and B). This suggests that during incubation, both *Bacillaceae* and *Clostridiaceae* (spore-forming Firmicutes) responded more rapidly to the changed conditions and proliferated, decreasing the detection of less abundant taxa. This is also reflected in the bacterial community composition (phyla and families Fig. 7A,B respectively) detected in the PAA-mann_free_ hydrogel system compared to the treated soil. Importantly their dominance is not seen in the PAA and PAA-mannan highlighting that polymer composition plays a clear role in bacterial community selection. This is supported by the PCO weighted UniFrac analysis (Fig. 6) showing the clustering of the different sample types. Here, it is noteworthy that although the difference in bacterial populations in the two hydrogel polymers had a low statistical significance, the PAA-mann_free_ samples grouped markedly closer together than the PAA, indicating a higher similarity in the community composition within the sample types, 78.3% similarity for PAA-mann_free_ and only 54.2% for PAA. Therefore the recruitment of bacteria from the surrounding soil by PAA-mann_free_ polymers seems to be more reliably selective compared to the PAA hydrogel.

### Functionality within the soil

The abundance and selectivity of bacteria within the PAA and PAA-mannan hydrogels relative to the soil in close proximity to a plant root would have the potential to both influence plant growth and be influenced themselves by root activity. Many of the taxa above identified by 16 S rRNA at the phylum and family level exemplify plant growth promoting functionality, where they may provide improved nutrient acquisition, production of plant hormones or plant disease suppression^35–37^. Additionally, microbial attachment to roots and formation of biofilms provide protection from environmental stresses such as pH and desiccation^38^. For example, Proteobacteria (Pr, Fig. 7A) can account for ~30% of dominant phyla in soil where they may provide nitrogen fixers, methane and ammonia oxiders as well as sulphide and sulphur users among their many members^39–42^. Firmicutes (Fi) engage various soil carbon sources, while Bacteroidetes (Ba) provide aerobic degradation of plant carbon material^43^. At the family level*, Chitinophagaceae* (Ch) has several species that can provide phosphate- and zinc-solubilizing ability enhancing overall soil fertility^44^, for example *Flavisolibacter ginsengiterrae* has been found to be highly associated with mannan residues^45^.

Figure 7 clearly shows consistency in the mannan induced selectivity between the model bacterial species and the soil microcosm communities. At the phylum level, the relative abundance Firmicutes (Fi) significantly declined in PAA-mannan whereas the Proteobacteria (Pr) increased greatly, consistent with model adhesin containing *Pseudomonas* spp. (*P. fluorescens)* (Fig. 2). While at the family level, this is exemplified by the marked and progressive reduction in *Bacillaceae* (Ba) abundance in PAA and PAA-mannan and the corresponding adhesin free model Bacillus (*B. subtilis)* which showed little difference in the ingress between PAA and PAA-mannan, illustrating that the single strain model was consistent with the behaviour of the complex soil bacterial community.

In conclusion, we show that the presence of mannans as either a copolymer or an interpenetrating network with the synthetic hydrogel PAA enhances the dynamics and selectivity of bacterial ingress, as shown by both model microbial systems and soil microcosms. Both PAA-mannan polymers maintained the original microstructure providing confining fluid environments on the scale of bacterial swimming motility and its associated hydrodynamic interactions. The presence of the additional specific interactions between mannan chains of the hydrogel and bacteria with adhesin proteins significantly increased ingress and colonization demonstrating selectivity. Incubation within microcosms of a wheat producing agricultural soil allowed the quantification of soil microbial ingress as well as selectivity of the polymer hydrogels with and without mannan chains. Hydrogel populations were significantly different from the corresponding soil after incubation, with all polymers having higher numbers than the incubated soil. Bacterial diversity was also significantly higher in mannan containg hydrogels in comparison to both synthetic PAA and the incubated soil resulting in a different microbial community between sample types. Importantly for some key microbial taxa, the hydrogels showed significantly enhanced selectivity compared to the positive control (no hydrogel) soil which was also consistent with the impact of adhesins on the selectivity of the model bacteria. Additionally, their osmotic potential maintains a hydrous state in comparison to surrounding soil undergoing drying. These data demonstrate that polymeric hydrogels, particularly those that exploit specific functional interactions with soil microbial communities, could play a significant role in mitigating biotic and abiotic stresses during the increasing intensity of climatic change. In doing so, functional polymeric materials may provide a new approach to address the global challenge of climate-limited crop productivity, especially when confined to the plant root developing zone.

## Materials and Methods

### Materials

Polyacrylic acid (PAA) and the corresponding mannan grafted polyacrylic acid (PAA-mann_graft_) were synthesized from anhydrous 99% acrylic acid containing monomethyl ether hydroquinone as inhibitor, 99% methylene bisacrylamide as crosslinker and ammonium persulphate as a water soluble initiator from Sigma Aldrich (Australia) according to Supporting Information S-1. The high molecular weight mannan, derived from *Saccharomyces cerevisiae* (Sigma Aldrich, M3640), is a linear chain of 1,4-linked β-D-mannopyranosyl residues having less than 5% of galactose^46,47^, was used without further purification. Mannan grafted PAA (PAA-mann_graft_) prepared according to S1.1 to yield 11.8 wt. % mannan in the dry hydrogel. PAA containing free mannan chains (PAA-mann_free_) was prepared by absorption from solution according to S1.1 to yield PAA with a 3.4 wt. % mannan content.

### Hydrogel characterization

The equilibrium swelling capacities (Q) of the respective hydrogels were determined with pre-weighed dry samples placed in cylindrical glass columns (ID 10 mm) supported by a hydrophilic nylon filter (11 μm pore size) to allow water permeation from below. Q values were then determined from the mass water uptake.

Viscoelastic micromechanical measurements of PAA and the PAA-mann hydrogels were performed with a stress controlled rheometer (DSR 200, Rheometrics, Paulsboro NJ) using vane geometry at 20 °C according to Barnes^48^. The vane geometry was adapted for swollen hydrogels to eliminate structural disturbance and wall-slip effects in the swollen gel state. Dehydration of samples occurring during measurements was minimised by placing the specimens in an environmental chamber at high humidity. Hydrogels were swollen within the rheometer assembly to Q values without disruption at vane surfaces prior to oscillatory stress measurements at frequency 0.1 rad/s and the viscoelastic parameters extracted.

The microstructure of the PAA and PAA-mannan hydrogels were determined by field emission cryo-SEM (FEI NOVA nanoSEM, FEI Co., Hillsboro, Oregon). Samples were prepared *in-situ* using the Gatan Alto 2500 pre-chamber followed by fracture to provide free-break surfaces which were then followed by low temperature sublimation, Pt sputtering and insertion in the cryo-SEM, as detailed previously^26^.

### Bacterial strains and growth conditions

*Bacillus subtilis* ATCC 6051 ^T^ and *Pseudomonas fluorescens* ATCC 13525 were obtained from the American Type Culture Collection (ATCC, USA). Bacterial stocks were prepared in nutrient broth (Oxoid) supplemented with 20% glycerol as previously described^49,50^. For each experiment, bacterial cultures were refreshed from stock on nutrient agar (Oxoid) and were collected at the logarithmic stage of growth. The cell density of these bacterial suspensions was adjusted to the optical density of 0.3 (λ at 600 nm) to ensure a consistent number of cells of each strain in all experiments.

Prior to incubation, hydrogels were swollen to equilibrium in either MilliQ water or nutrient broth at an appropriate concentration. Pre-swollen hydrogels were then immersed in 10 mL of bacterial suspension and incubated at 25 °C for either 18 or 48 hours. After incubation, the samples were gently removed from the suspension followed by a mild rinsing step to remove unbound bacteria without disturbing the bacterial colonization within the hydrogels. All experiments were repeated at least three times (each with duplicates) independently.

### Quantification of bacterial ingress

The extent of bacterial ingress and colonization into the three dimensional network of hydrogels was studied using confocal laser scanning microscopy (CLSM). The specimen was stained with LIVE/DEAD BacLight Bacterial Viability Kit as described previously^26^. After the staining process, the specimen was imaged using an inverted confocal laser scanning microscope (Fluoview FV1000- IX81, Olympus, Japan). To determine the distance of bacterial ingress within the hydrogel network, depth scans (z-stack) were acquired up to 200 µm starting from the interface of the hydrogel. All three dimensional stacks were presented in the vertical plane (x-z) together with the integrated fluorescence intensity of both viable and non-viable cells, to detail the differences in cell behaviour inside the hydrogels as well as the depth of cell penetration (advancing microbial front). The location of the biointerfaces of hydrogel samples was identified and the staining-washing procedure of the bacteria-colonized hydrogels was validated as previously reported^26^. The number of colonized bacterial cells per field of view (xyz is approximately 211 µm × 211 µm × 200 µm respectively) was calculated using ImageJ with a 3D Objects Counter plugin^51^. The velocity of *B. subtilis* swimming cells was determined by calculating the total time and distance a single cell travelled within a field of view, appearing as the “cell trajectory” in the 2D x-z plane.

### Soil microbe population and taxa selectivity

Soil microcosms were established using soil collected from the surface (0–10 cm) near Dandaragan in the central agricultural (wheat belt) of Western Australia (S 30°51′57.24″, E 115°42′39.45″). Soil was naturally air-dried at 40 °C and sieved (2 mm) before construction of soil microcosms. Microcosms consisted of dry soil (7.5 g) added to 15 mL Falcon tubes. Where applicable, a single, dry polymer granule was then added and covered with an additional 7.5 g of soil and compacted to a bulk density of 1.4 g cm^−3^. Fifteen microcosms were established containing: 5 with soil only, 5 with PAA, and 5 with a PAA-mann_free_ granules. To wet each of these microcosms, 4 mL of water were added to the surface and allowed to infiltrate. This resulted in a soil water content of 26.7% well below the previously determined water-holding capacity of 31.2% of the soil excluding ponding of water at the bottom of the tube. This relatively high soil moisture, reflecting soil after rainfall events, was required to ensure complete swelling of the polymer system.

After one week of incubation at 25 °C in the dark, microcosms were dismantled and DNA was extracted from the soil and polymers respectively using the MO BIO PowerSoil® DNA Isolation Kit (Carlsbad, CA, USA) and quantified using the Qubit® 2.0 Fluorometer (ThermoFisher Scientific, Waltham, MA,USA) following manufacturers’ protocols. Genomic DNA was submitted to the Australian Genome Research Facility for diversity profiling of the 16 S rRNA gene using primers 341 F (5′-CCTAYGGGRBGCASCAG-3′) and 806 R (5′-GGACTACNNGGGTATCTAAT-3′)^52^. Paired-ends reads were assembled by aligning the forward and reverse reads using PEAR (version 0.9.5)^53^. Primers were then identified and trimmed. Trimmed sequences were processed using Quantitative Insights into Microbial Ecology (QIIME 1.8)^54^ and USEARCH (version 8.0.1623) software^55^. Chimera were removed and operational taxonomic units were picked with uclust^55^. Taxonomy was assigned using the SILVA database^56^. OTUs were rarefied to 51,367 sequences per sample prior to conducting statistical analyses of bacterial community compositions using PRIMER-E (version 7). PERMANOVA and SIMPER^57^ were performed on log-transformed relative abundance data^58^, while PCoA plots were based on unweighted UniFrac distance metrics^59^.

Quantitative PCR to determine the abundance of 16 S rRNA genes was conducted with a ViiA7 qPCR system (Applied Biosystems, ThermoFisher). qPCR reactions were carried out in 20 μL containing: 10 μL of GoTaq^®^ qPCR Master Mix (Promega), 0.1 μl of the forward (Eub338 5′ ACTCCTACGGGAGGCAGCAG 3′)^60^ and reverse primer (Eub518 5′ ATTACCGCGGCTGCTGG 3′)^61^ at 20 μM, 2 μl BSA at a concentration of 50 µg µL^−1^ (Ultrapure BSA, Ambion); 2 μl of template DNA at 1 ng μL^−1^ and 5.8 μL of water. Cycling conditions were 95 °C for 15 min then 40 cycles of 95 °C for 60 s, 53 °C for 30 s and 72 °C for 60 s^62^. Fluorescence data were collected at 72 °C to confirm product specificity and melting curve analyses were conducted after each cycle. Standard curves were generated using dilutions of linearised cloned plasmids containing the target 16 S rRNA gene sequence. Standard curves generated in each reaction were linear over five orders of magnitude (10^5^ to 10^9^ gene copies) with r^2^ values greater than 0.99, and efficiencies for all quantification reactions were 90% to 100%. Abundance data were analysed using one-way ANOVA and Tukey Honestly Significant Difference tests.

### Data Availability

Sequencing data from this study are available on Qiita under study ID 11154 (https://qiita.ucsd.edu/study/description/11154).

## Electronic supplementary material

Supplementary information

## References

[1] Caló E, Khutoryanskiy VV (2015). Biomedical applications of hydrogels: A review of patents and commercial products. Eur. Polym. J..

[2] Schoebitz M, López MD, Roldán A (2013). Bioencapsulation of microbial inoculants for better soil–plant fertilization. A review. Agron. Sustain. Dev..

[3] Veiga AS, Schneider JP (2013). Antimicrobial hydrogels for the treatment of infection. Biopolymers.

[4] Yan Z, Shi P, Ren J, Qu XA (2015). “sense-and-treat” hydrogel used for treatment of bacterial infection on the solid matrix. Small.

[5] Kolewe KW, Peyton SR, Schiffman JD (2015). Fewer bacteria adhere to softer hydrogels. ACS Appl. Mater. Interfaces.

[6] Kearns DB (2010). A field guide to bacterial swarming motility. Nature Rev. Microbiol..

[7] Classen AT (2015). Direct and indirect effects of climate change on soil microbial and soil microbial-plant interactions: What lies ahead?. Ecosphere.

[8] Timmusk S (2014). Drought-tolerance of wheat improved by rhizosphere bacteria from harsh environments: Enhanced biomass production and reduced emissions of stress volatiles. PLoS ONE.

[9] Bernardi A (2013). Multivalent glycoconjugates as anti-pathogenic agents. Chem. Soc. Rev..

[10] Xue X (2011). Synthetic polymers for simultaneous bacterial sequestration and quorum sense interference. Angew. Chem. Int. Ed..

[11] Tuson HH (2012). Measuring the stiffness of bacterial cells from growth rates in hydrogels of tunable elasticity. Mol. Microbiol..

[12] Song F, Ren D (2014). Stiffness of cross-linked poly(dimethylsiloxane) affects bacterial adhesion and antibiotic susceptibility of attached cells. Langmuir.

[13] Jarrell KF, McBride MJ (2008). The surprisingly diverse ways that prokaryotes move. Nature Rev. Microbiol..

[14] Eric L, Thomas RP (2009). The hydrodynamics of swimming microorganisms. Rep. Prog. Phys..

[15] Normand T, Lauga E (2008). Flapping motion and force generation in a viscoelastic fluid. Phys. Rev. E.

[16] Trouilloud R, Yu TS, Hosoi AE, Lauga E (2008). Soft swimming: Exploiting deformable interfaces for low-Reynolds number locomotion. Phys. Rev. Lett..

[17] Dechesne A, Wang G, Gulez G, Or D, Smets BF (2010). Hydration-controlled bacterial motility and dispersal on surfaces. Proc. Natl. Acad. Sci. USA.

[18] Long T, Or D (2005). Aquatic habitats and diffusion constraints affecting microbial coexistence in unsaturated porous media. Water Resour. Res..

[19] Treves DS, Xia B, Zhou J, Tiedje JM (2003). A two-species test of the hypothesis that spatial isolation influences microbial diversity in soil. Microb. Ecol..

[20] Ofek I, Sharon N (1988). Lectinophagocytosis: a molecular mechanism of recognition between cell surface sugars and lectins in the phagocytosis of bacteria. Infect. Immun..

[21] Pieters RJ (2007). Intervention with bacterial adhesion by multivalent carbohydrates. Med. Res. Rev..

[22] Vesper SJ (1987). Production of pili (fimbriae) by *Pseudomonas fluorescens* and correlation with attachment to corn roots. Appl. Environ. Microbiol..

[23] Berne, C., Ducret, A., Hardy, G. G. & Brun, Y. V. Adhesins involved in attachment to abiotic surfaces by Gram-negative bacteria. *Microbiol. Spectr*. **3** (2015). 10.1128/microbiolspec.MB-0018-2015 PMC4566860 26350310

[24] Augimeri RV, Varley AJ, Strap JL (2015). Establishing a role for bacterial cellulose in environmental interactions: Lessons learned from diverse biofilm-producing Proteobacteria. Front. Microbiol..

[25] Zinger-Yosovich K, Sudakevitz D, Imberty A, Garber NC, Gilboa-Garber N (2006). Production and properties of the native *Chromobacterium violaceum* fucose-binding lectin (CV-IIL) compared to homologous lectins of *Pseudomonas aeruginosa* (PA-IIL) and *Ralstonia solanacearum* (RS-IIL). Microbiology.

[26] Truong VK, Mainwaring DE, Murugaraj P, Nguyen DHK, Ivanova EP (2015). Impact of confining 3-D polymer networks on dynamics of bacterial ingress and self-organisation. J. Mater. Chem. B.

[27] Horkay F, Tasaki I, Basser PJ (2000). Osmotic swelling of polyacrylate hydrogels in physiological salt solutions. Biomacromolecules.

[28] Savina IN (2011). Porous structure and water state in cross-linked polymer and protein cryo-hydrogels. Soft Matter.

[29] Li, M. *Experimental study of swimming flagellated bacteria and their collective behaviour in concentrated suspensions*, University of Edinburgh (2010).

[30] Cisneros LH, Cortez R, Dombrowski C, Goldstein RE, Kessler JO (2007). Fluid dynamics of self-propelled microorganisms, from individuals to concentrated populations. Exp. Fluids.

[31] Solari CA, Kessler JO, Goldstein RE (2007). Motility, mixing, and multicellularity. Genet. Program. Evol. M..

[32] Sato Y, Kubo T, Morimoto K, Yanagihara K, Seyama T (2016). High mannose-binding *Pseudomonas fluorescens* lectin (PFL) downregulates cell surface integrin/EGFR and induces autophagy in gastric cancer cells. BMC Cancer.

[33] Sato Y, Morimoto K, Kubo T, Yanagihara K, Seyama T (2012). High mannose-binding antiviral lectin (PFL) from *Pseudomonas fluorescens* Pf0-1 promotes cell death of gastric cancer cell MKN28 via interaction with α2-integrin. PLoS ONE.

[34] Kunst F (1997). The complete genome sequence of the Gram-positive bacterium *Bacillus subtilis*. Nature.

[35] Kloepper JW, Leong J, Teintze M, Schroth MN (1980). Enhanced plant growth by siderophores produced by plant growth-promoting rhizobacteria. Nature.

[36] Kloepper JW, Lifshitz R, Zablotowicz RM (1989). Free-living bacterial inocula for enhancing crop productivity. Trends Biotechnol..

[37] Glick BR (1995). The enhancement of plant growth by free-living bacteria. Can. J. Microbiol..

[38] Swarnalakshmi K (2013). Evaluating the influence of novel cyanobacterial biofilmed biofertilizers on soil fertility and plant nutrition in wheat. Eur. J. Soil Biol..

[39] Fierer N, Bradford MA, Jackson RB (2007). Toward an ecological classification of soil bacteria. Ecology.

[40] Janssen PH (2006). Identifying the dominant soil bacterial taxa in libraries of 16S rRNA and 16S rRNA genes. Appl. Environ. Microb..

[41] Nold SC, Zhou J, Devol AH, Tiedje JM (2000). Pacific northwest marine sediments contain ammonia-oxidizing bacteria in the β subdivision of the Proteobacteria. Appl. Environ. Microbiol..

[42] Chen W-M (2003). Legume symbiotic nitrogen fixation by β-Proteobacteria is widespread in nature. J. Bacteriol..

[43] Goldfarb KC (2011). Differential growth responses of soil bacterial taxa to carbon substrates of varying chemical recalcitrance. Front. Microbiol..

[44] Chung EJ, Park TS, Jeon CO, Chung YR (2012). *Chitinophaga oryziterrae* sp. nov., isolated from the rhizosphere soil of rice (Oryza sativa L.). Int. J. Syst. Evol. Microbiol..

[45] Yoon MH, Im WT (2007). *Flavisolibacter ginsengiterrae* gen. nov., sp. nov. and *Flavisolibacter ginsengisoli* sp. nov., isolated from ginseng cultivating soil. Int. J. Syst. Evol. Microbiol..

[46] Moreira LR, Filho EX (2008). An overview of mannan structure and mannan-degrading enzyme systems. Appl. Microbiol. Biotechnol..

[47] Nakajima T, Ballou CE (1974). Characterization of the carbohydrate fragments obtained from *Saccharomyces cerevisiae* mannan by alkaline degradation. J. Biol. Chem..

[48] Barnes HA (1999). The yield stress—a review or ‘παντα ρει’—everything flows?. J. Non-Newton Fluid.

[49] Truong VK (2015). Self-organised nanoarchitecture of titanium surfaces influences the attachment of *Staphylococcus aureus* and *Pseudomonas aeruginosa* bacteria. Appl. Microbiol. Biotechnol..

[50] Ivanova EP (2011). Differential attraction and repulsion of *Staphylococcus aureus* and *Pseudomonas aeruginosa* on molecularly smooth titanium films. Sci. Rep..

[51] Bolte S, Cordelieres FP (2006). A guided tour into subcellular colocalization analysis in light microscopy. J. Microsc..

[52] Caporaso JG (2011). Global patterns of 16S rRNA diversity at a depth of millions of sequences per sample. Proc. Natl. Acad. Sci. USA.

[53] Zhang J, Kobert K, Flouri T, Stamatakis A (2014). PEAR: a fast and accurate Illumina Paired-End reAd mergeR. Bioinformatics.

[54] Caporaso JG (2010). QIIME allows analysis of high-throughput community sequencing data. Nat. Methods.

[55] Edgar RC (2010). Search and clustering orders of magnitude faster than BLAST. Bioinformatics.

[56] Quast C (2013). The SILVA ribosomal RNA gene database project: improved data processing and web-based tools. Nucleic Acids Res..

[57] Hirsch PR (2017). Soil resilience and recovery: rapid community responses to management changes. Plant Soil.

[58] Clarke, K. & Gorley, R. PRIMERv7: User manual/tutorial. PRIMER-E, Plymouth, 296pp. (2015).

[59] Lozupone C, Knight R (2005). UniFrac: a new phylogenetic method for comparing microbial communities. Appl. Environ. Microbiol..

[60] Goodfellow, M. & Stackebrandt, E. *Nucleic acid techniques in bacterial systematics* Vol. 5 (John Wiley & Sons Ltd, 1991).

[61] Muyzer G, de Waal EC, Uitterlinden AG (1993). Profiling of complex microbial populations by denaturing gradient gel electrophoresis analysis of polymerase chain reaction-amplified genes coding for 16S rRNA. Appl. Environ. Microbiol..

[62] Fierer N, Jackson JA, Vilgalys R, Jackson RB (2005). Assessment of soil microbial community structure by use of taxon-specific quantitative PCR assays. Appl. Environ. Microbiol..

