# Supplementary material for: Copolymers enhance selective bacterial community colonization for potential root zone applications
Source: Sci Rep. 2017 Nov 21;7:15902. doi: 10.1038/s41598-017-16253-0 (PMC5698314; doi:10.1038/s41598-017-16253-0)
Supplement: Supplementary file 1 — Supplementary information [file 41598_2017_16253_MOESM1_ESM.pdf]

# Supplementary Material

## Copolymers enhance selective bacterial community colonization for potential root zone applications

Vy T.H. Pham[1], Pandiyan Murugaraj[1], Falko Mathes[2], Boon K.Tan[1], Vi Khanh Truong[1], Daniel V. Murphy[2], David E. Mainwaring[1,*]

[1]School of Science, Faculty of Science, Engineering and Technology, Swinburne University of Technology, Hawthorn VIC 3122, Australia

[2]SoilsWest, UWA School of Agriculture and Environment, Faculty of Science, The University of Western Australia, Crawley, WA6009, Australia

[*]Corresponding author: Email to demainwaring@swin.edu.au

## Materials & methods

**S1 Synthesis of polyacrylic acid (PAA) and mannan grafted polyacrylic acid ($PAA_{graft.}$).** Polyacrylate (PAA) hydrogel was synthesized by reacting a mixture of acrylic acid (4.76 mL) pre-neutralised with KOH and methylene bisacrylamide as the crosslinker (0.011 g in 2 mL of water) at 85 °C under $N_2$ prior to the addition of ammonium persulphate initiator (0.042 g in 2 ml of water). Gelation occurred over a 2 hour period. The resultant PAA had a degree of neutralisation (DN) of 75 % and degree of crosslinking 0.066. A corresponding mannan grafted polyacrylate hydrogel ($PAA\text{-}mann_{graft.}$) was synthesized by reacting a mixture of pre-neutralised acrylic acid (4.76 mL) with an aqueous solution of mannan (0.58 g in 15 mL water) and methylene bisacrylamide (0.011 g in 2 mL of water) at 85 °C under $N_2$ prior to the addition of ammonium persulphate initiator (0.042 g in 2 ml of water), as indicated schematically below. Following both the PAA and $PAA\text{-}mann_{graft.}$ syntheses, the resulting products were cleaned to remove unreacted monomers and other impurities by repeated swelling in milliQ water followed by de-swelling in ethanol (4 cycles) prior to drying at 80 °C. The freely adsorbed mannan counterpart ($PAA\text{-}mann_{free}$) was prepared by swelling 55mg of dried PAA in 6.8 mL of mannan solution (0.275 mg/mL) until all the mannan solution was fully absorbed prior to drying at the lower temperature of 60 °C.

**S2 Hydrogel properties.** Equilibrium swelling capacity of the hydrogel was determined where pre-weighed dry hydrogels were placed in a cylindrical glass column (ID 10 mm) supported by a hydrophilic nylon filter (11 µm pore size) to allow water permeation from below. The swelling ratios (Q values) were then determined from the equilibrium mass water uptake.

## Results

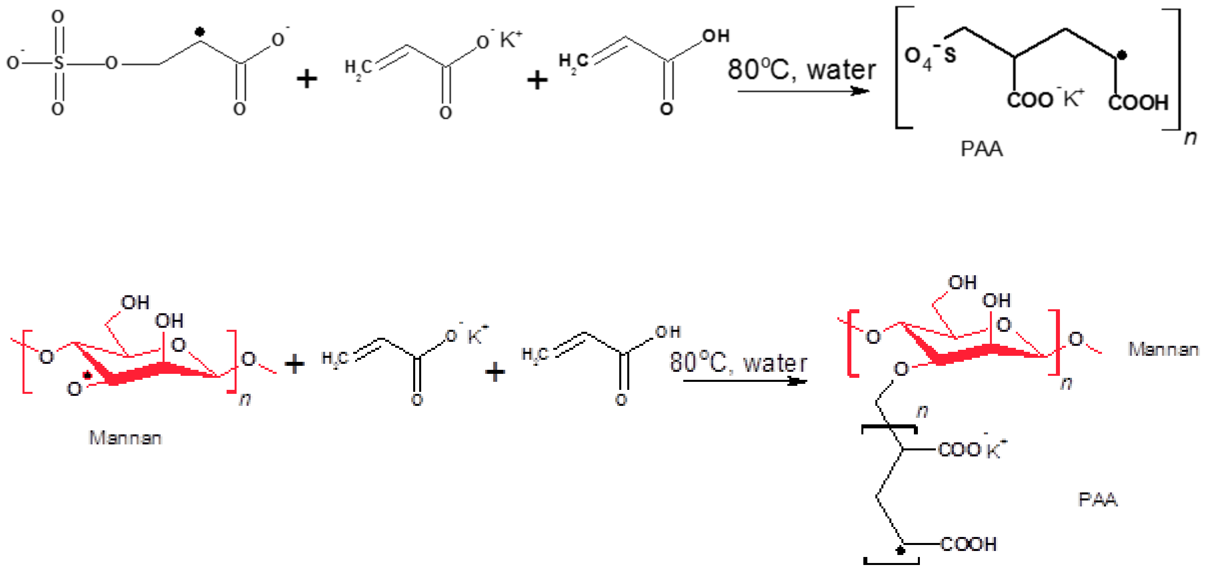


**Supplementary Figure 1 | Synthesis scheme generating hydrogel polymer and co-polymers.** The free radical on the acrylate molecule was readily reacted to form polyacrylic acrylate (PAA) chains via the unsaturated π-bond of the acrylic acid and K-acrylate monomers.[1,2] Similarly the free radical on the mannan molecule may attack the unsaturated π-bond of the acrylic acid and K-acrylate monomers to form mannan-grafted PAA chain. During the polymerization, the free radicals on both PAA chains and mannan grafted-PAA chains react with the cross-linker (MBA) molecules to further form the hydrogel network.

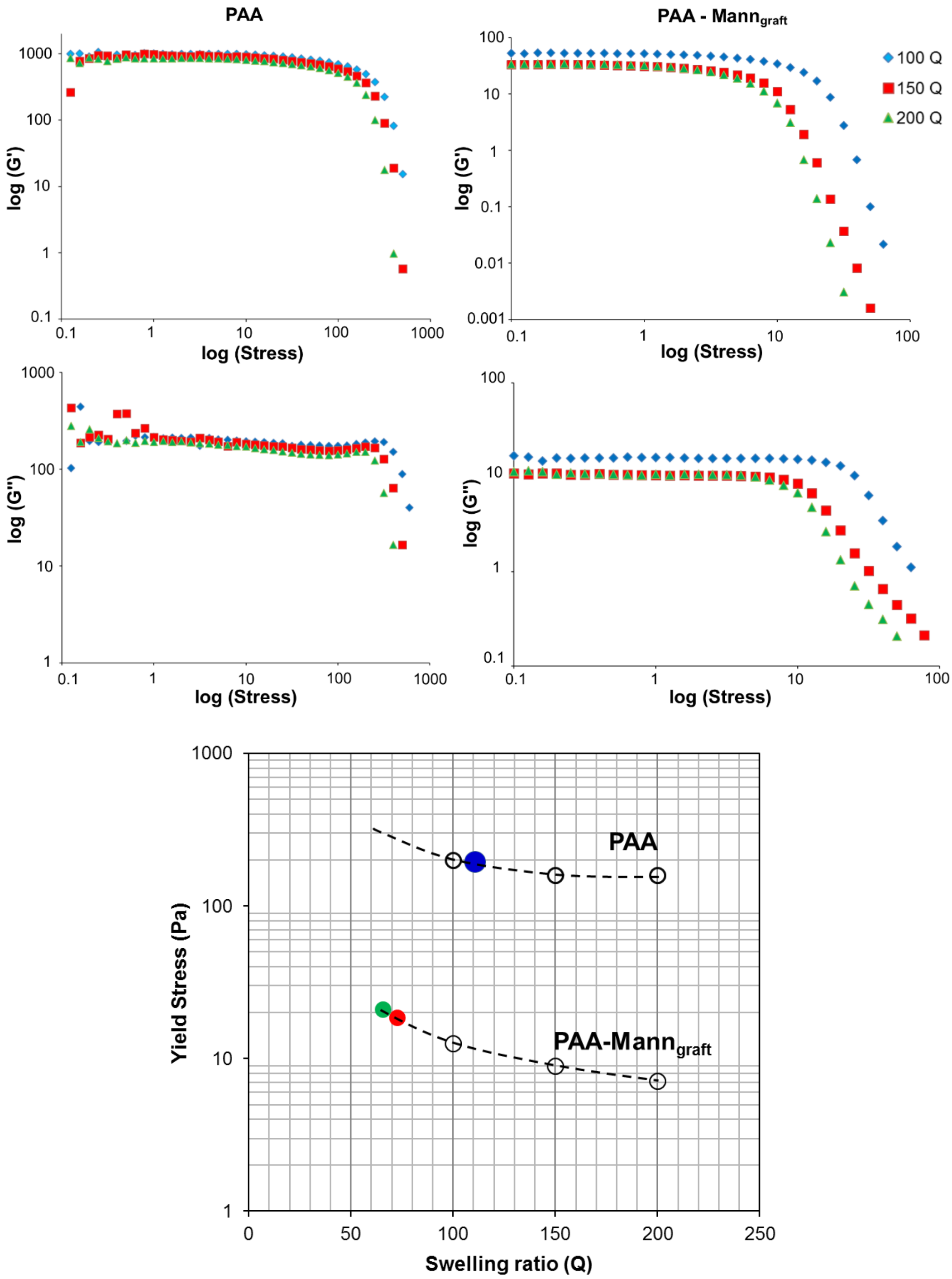


**Supplementary Figure 2 | Structural properties of hydrated hydrogels.** The viscoelastic behaviour of PAA and PAA-mann$_{graft}$ hydrogels obtained from undisturbed swollen hydrogel with the vane geometry and the rigidity of the polymer hydrogels with increasing water uptake. Coloured symbols represent the polymer hydrogels used in this study (blue: PAA, green: PAA-mann$_{graft}$, red: PAA-mann$_{free}$). Dotted lines added to guide the eye.

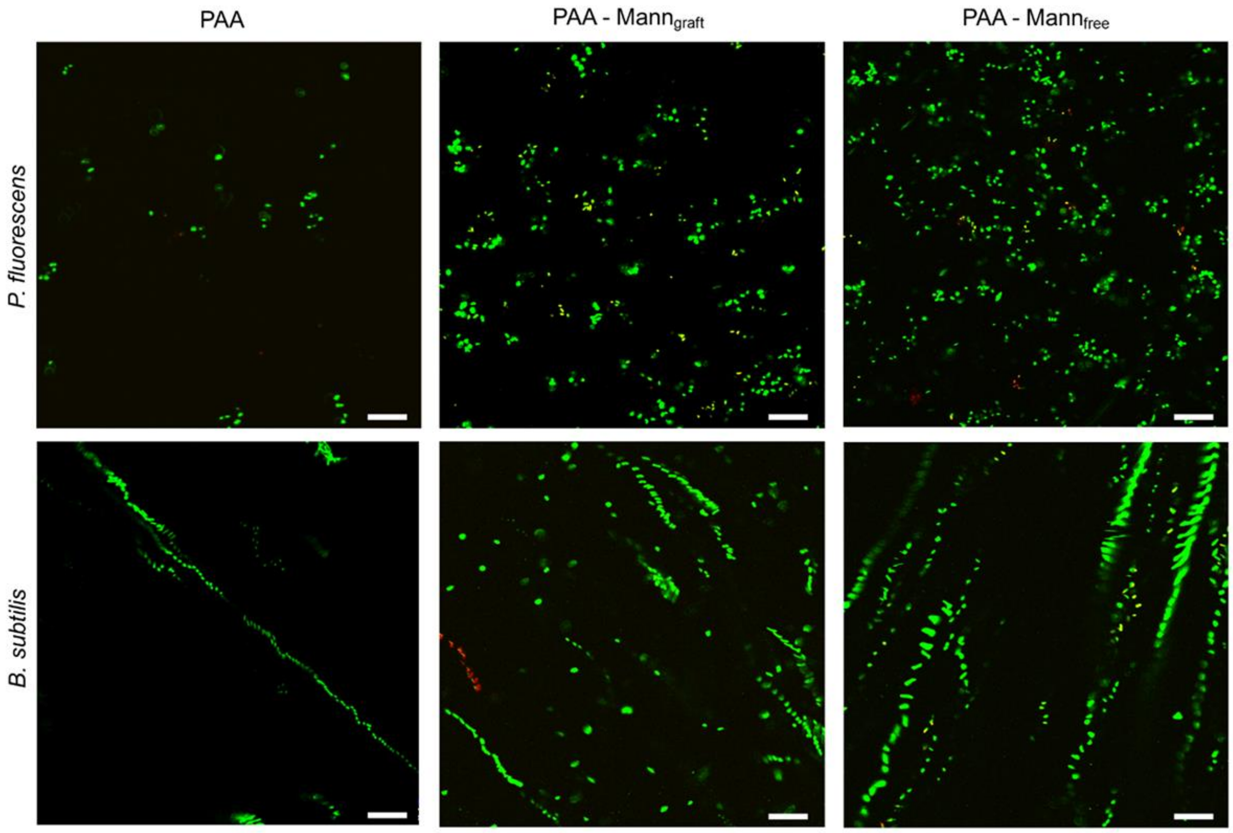


**Supplementary Figure 3 | Microbial motility and colonization within hydrogels after 18 hour.** Differential behaviour of *Pseudomonas fluorescens* and *Bacillus subtilis* bacterial cells within the inner bulk region of hydrogels after 18 hours. *P. fluorescens* appeared to form micro-colonies in association with the polymeric chains, while *B. subtilis* cells were seen to be highly motile. Green indicates viable cells (stained with SYTO 9), while red indicates damaged cells (stained with propidium iodide). Scale bar is 25 µm.

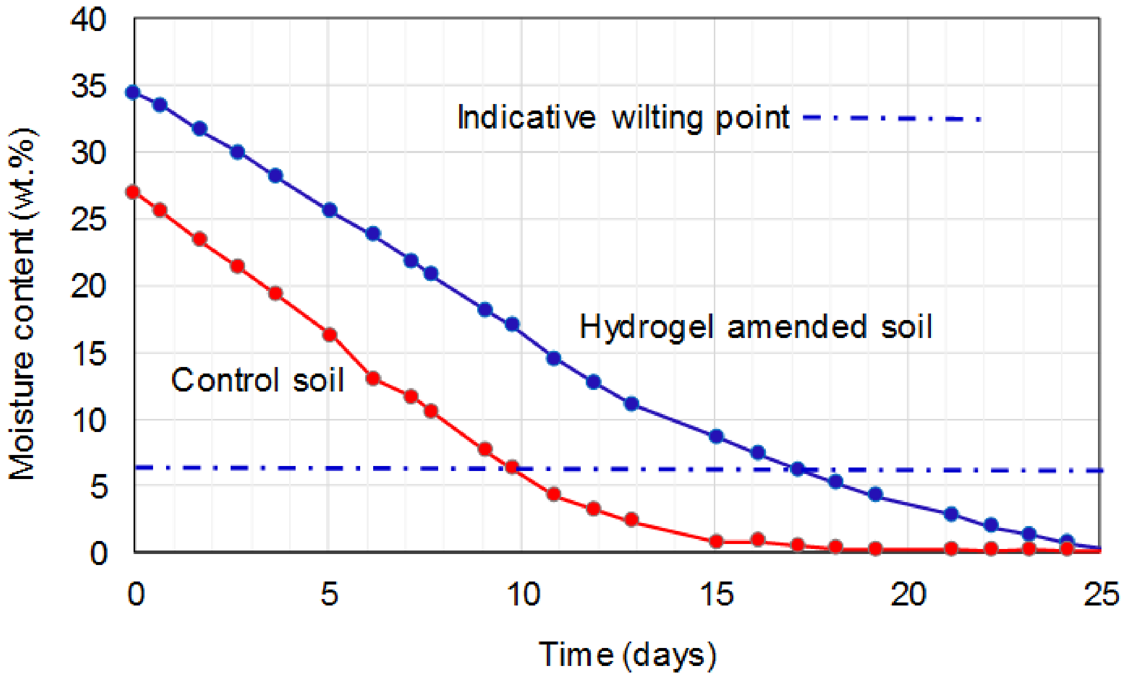


**Supplementary Figure 4 | Impact of PAA hydrogel on soil moisture during drying.** Comparison of moisture content in the region of a model rhizosphere with and without hydrogel amendment and where the mass ratio of soil to PAA was 500 / 1. Over the bulk of the drying, approximately 10% additional moisture was retained, indicative of a hydrogel water content (Q) about 50 g water / g PAA.

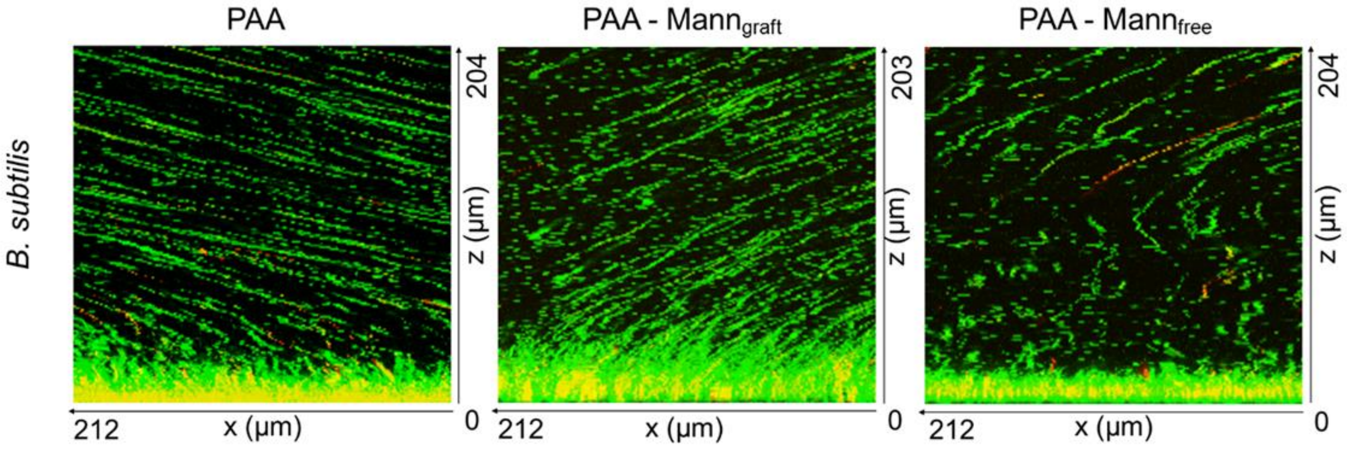


**Supplementary Figure 5 | Bacterial ingress into hydrogels after 48 hour.** The ingress of *Bacillus subtilis* cells into the hydrogels after 48 hour incubation indicates that overall population increased with ingress time but the relative population profiles were not dominated by time dependence.

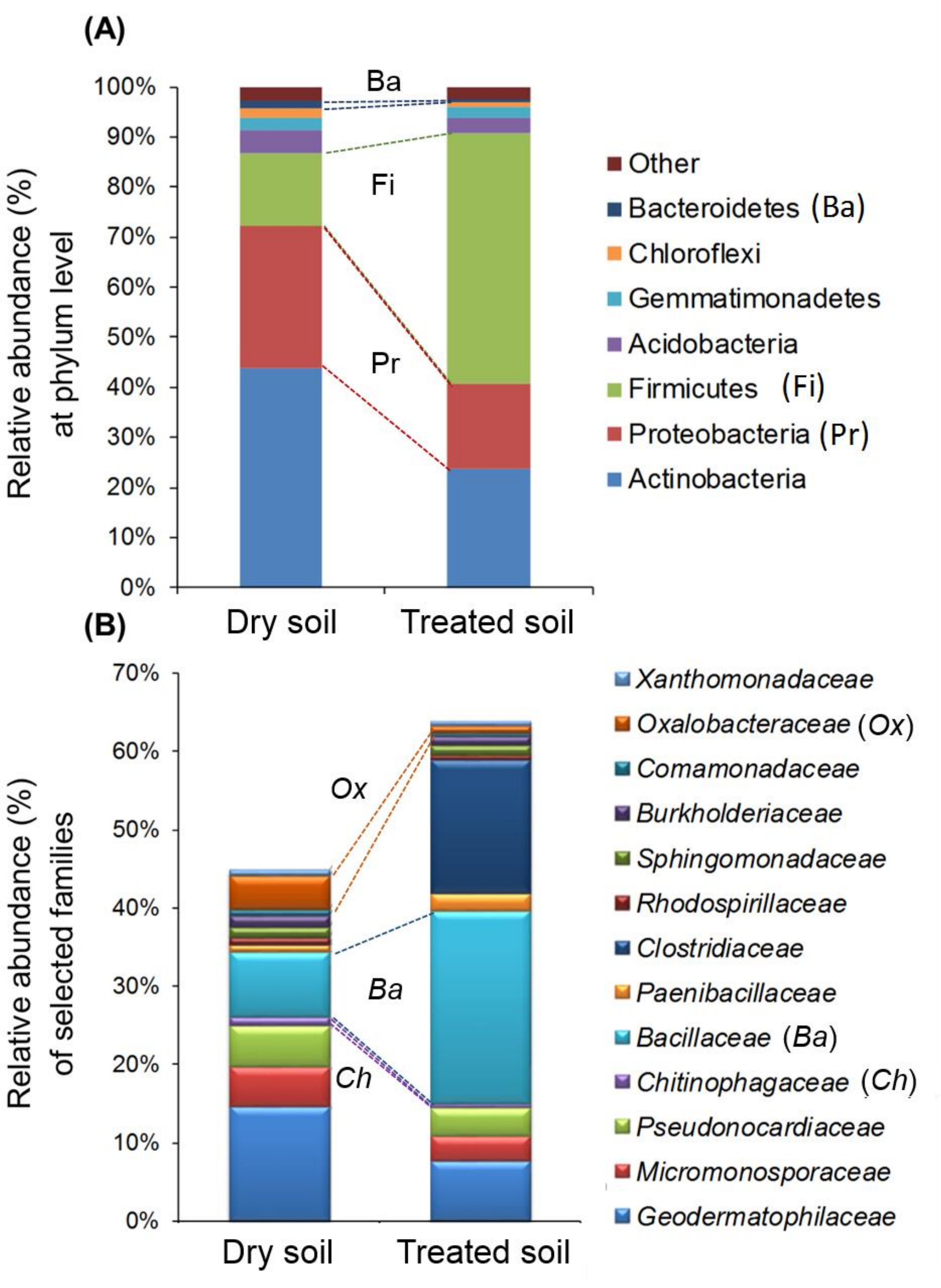


**Supplementary Figure 6│Relative composition of bacterial community in soil microcosms after 7 days.** Relative abundance of the most representative phyla (A) and families (B) present within the microcosm of the original dry Dandaragan soil compared the soil incubated for 7 days at 25°C, as seen by 16S rRNA gene sequencing (n = 5).

## References


1. Pourjavadi, A., Harzandi, A. M. & Hosseinzadeh, H. Modified carrageenan 3. Synthesis of a novel polysaccharide-based superabsorbent hydrogel via graft copolymerization of acrylic acid onto kappa-carrageenan in air. *Eur. Polym. J.* **40**, 1363-1370 (2004).
2. Roy, D., Semsarilar, M., Guthrie, J. T. & Perrier, S. Cellulose modification by polymer grafting: A review. *Chem. Soc. Rev.* **38**, 2046-2064 (2009).
